# Hooked on fish blood: the reliance of a gill parasite on haematophagy

**DOI:** 10.1098/rspb.2024.1611

**Published:** 2024-10-30

**Authors:** Enrique Riera-Ferrer, Itziar Estensoro, Beatriz López-Gurillo, Raquel Del Pozo, Francisco Esteban Montero, Ariadna Sitjà-Bobadilla, Oswaldo Palenzuela

**Affiliations:** ^1^ Fish Pathology Group, Institute of Aquaculture Torre de la Sal (IATS, CSIC), Consejo Superior de Investigaciones Científicas, Castellón, Spain; ^2^ Cavanilles Institute for Biodiversity and Evolutionary Biology, Science Park, University of Valencia, Valencia 46071, Spain

**Keywords:** monogenea, EDX-TEM, gastrodermis, haemoglobin, iron, buccal complex

## Abstract

Parasitism involves diverse evolutionary strategies, including adaptations for blood feeding, which provides essential nutrients for growth and reproduction. *Sparicotyle chrysophrii* (Polyopisthocotyla: Microcotylidae), an ectoparasitic flatworm, infects the gills of gilthead seabream (*Sparus aurata*), significantly affecting fish health, welfare and Mediterranean cage farm profitability. Despite its impact, limited information exists on its feeding behaviour. This study demonstrates the presence of blood and exogenous haem groups in *S. chrysophrii* and explores its digestive tract using light and electron microscopy, elucidating its internal morphology and spatial arrangement. Elemental analysis of the digestive haematin cells shows residual oxidized haem depots as haematin crystals. Additionally, we studied the impact of the blood feeding on the host by estimating the average volume of blood intake for an adult parasite (2.84 ± 2.12µl·24h^–1^) and we described the significant drop of the plasmatic free iron levels in infected hosts. Overall, we demonstrate the parasite’s reliance on its host blood, the parasite’s buccal and digestive morphological adaptations for blood feeding and the provoked effect on the fish host's health.

## 1. Introduction

Haematophagy has evolved multiple times as a nutritional strategy associated with a parasitic lifestyle. Blood provides a rich source of carbohydrates for the production of energy and amino acids required for growth and reproduction purposes [1]. However, obligate haematophagy encompasses several functional and metabolic challenges to overcome. One of the main constraints threatening blood-feeding parasites is haemoglobin digestion, as the resulting free haem is highly toxic owing to its pro-oxidant nature and causes tissue damage and protein degradation. Among other effects, free radicals induced by haem interfere with phospholipid bilayer stability and accelerate cytolysis [2]. Consequently, haematophagous organisms have developed efficient haem-detoxification and storage routes.

Haematophagous organisms affecting terrestrial animals are diverse and include obligate and facultative parasites as well as micropredators, from the class Insecta Linnaeus, 1758 to the class Arachnida Lamarck, 1801. Among the phylum Annelida Lamarck, 1809, haematophagy is common in leeches (Hirudinia Lamarck, 1818) and has also been described in several helminths. Aquatic animals and fish are also infected by haematophagous parasites, such as some copepods, isopods, branchiurans and polyopisthocotylans, that lead health and welfare concerns in freshwater and marine aquaculture regimes.

Monogeneans, formerly considered a platyhelminth class (Monogenea Carus, 1863) within Neodermata Ehlers, 1985 and recently reclassified into two independent classes, Monopisthocotyla Brabec, Salomaki, Kolísko, Scholz, Kuchta, 2023 and Polyopisthocotyla Brabec, Salomaki, Kolísko, Scholz, Kuchta, 2023 [3,4], are mainly ectoparasites in aquatic environments. Some of them have a major impact on fish health and welfare. Infections by these parasites can cause tissue disruption, anaemia and respiratory and osmotic distress, and they often trigger secondary infections and increased fish mortality [5,6]. In the aquaculture industry, some monogeneans cause significant economic losses [5,7–10] including *Sparicotyle chrysophrii* Van Beneden and Hesse, 1863 in gilthead seabream (*Sparus aurata* Linnaeus, 1758) [11]. In terms of pathogenicity, the scientific literature is dominated by the class Polyopisthocotyla, most probably owing to their large size, prolificacy and presumed haematophagous lifestyle [12].

In *S. aurata* aquaculture industry, *S. chrysophrii* is considered by far the most devastating pathogen across the Mediterranean Sea. Up to 30% of the mortality during the on-growing culture in sea cages has been attributed to this parasitic flatworm [11,13], and its management implies extensive use of resources and manpower devoted to parasite monitoring and application of chemical treatments when infection intensity thresholds are reached. Infections by *S. chrysophrii* and by other polyopisthocotylans have been linked to severe host anaemia, locally reflected in pale gills, and it is generally accepted these parasites are haematophagous. However, direct evidence of blood feeding by these parasites is scarce [12,14] and this has not been demonstrated for *S. chrysophrii*, yet.

Unlike haematophagous arthropods, polyopisthocotylans lack any true piercing anatomical appendages. Instead, ultrastructure studies have described that the anterior end in these flatworms presents a buccal complex consisting of a pair of buccal suckers, a mouth cavity, a pharynx, a putative taste organ and associated structures such as glandular ducts, and a valve apparatus in some species [15,16]. The pair of buccal suckers are strongly muscular, each anatomically situated within a buccal capsule, probably allowing the protrusion of the suckers, improving their attachment to the host and sealing, and with their lumina connecting to the mouth cavity [15–17]. The access to host blood would be facilitated by the secretion of histolytic enzymes through the glandular ducts and the mouth cavity, and the blood intake would be regulated by the pharynx/valve apparatus [15–18]. Given the scarce functional studies on blood ingestion by polyopisthocotylans, it remains unclear to what extent the observed host anaemia can be attributed to haematophagy or the microhaemorrhages caused by mechanical injuries from the parasite’s attachment organ, known as the haptor.

Haem and iron are crucial for growth and reproduction of many organisms. Moreover, vitellogenins are primary constituents of yolk proteins in eggs and have been described as iron-binding proteins related to haem detoxification and embryogenesis in haematophagous parasitic and micropredator arthopods [19,20]. Similarly, yolk ferritins were identified in *Clonorchis sinensis* Cobbold, 1875 [21] and *Schistosoma mansoni* Sambon, 1907 [22], and later it was determined that haem is essential for *S. mansoni* egg production [23]. However, free-living flatworms were described to have specific yolk ferritins that supply aluminium rather than iron to the vitellaria [24].

The focus of the current study encompasses the direct demonstration and the quantification of host blood intake, a detailed study of the parasite´s buccal complex, digestive tract and the elemental composition of its digestive cells. The investigation further reveals exogenous haem groups, the close association of iron with vitelline cells and vitellogenin, and assesses the parasite’s impact on the host’s plasmatic free iron levels.

## 2. Material and methods

### (a) *In vivo* maintenance of *Sparicotyle chrysophrii* in *Sparus aurata*

*Sparus aurata* were experimentally exposed to *S. chrysophrii* in the Fish Pathology facilities at the Institute of Aquaculture Torre de la Sal (IATS) located at 40°5 ´N; 0°10 ´E. Details of the *in vivo* parasite maintenance setup were previously described in Riera-Ferrer *et al*. [25]. Infected fish were used as parasite source in the subsequent assays.

### (b) Haemochromogen crystal assay—parasite *intra vitam* observations

**Figure 1.**
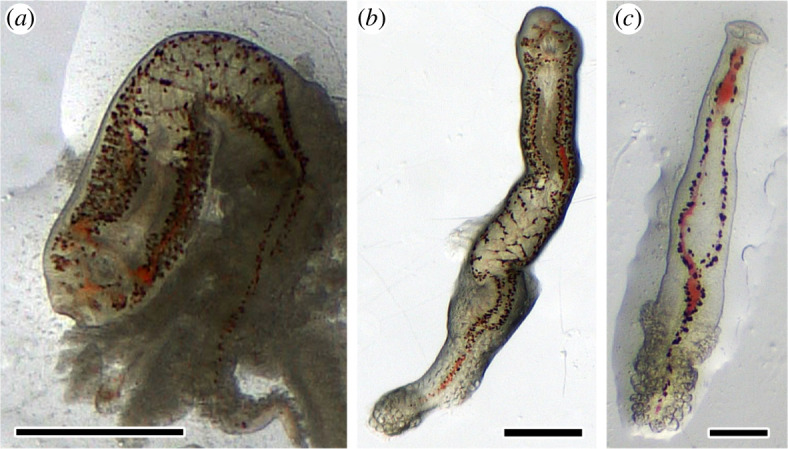
(*a*)–(*c*) Stereomicroscope images of different fresh *Sparicotyle chrysophrii* adult stages with internalized fresh bloodmeal. Note bloodmeal in different locations, indicative of the parasites’ digestive tract. Scale bars = 500 µm.

An infected host was euthanized by tricaine methanesulfonate (MS-222; Sigma-Aldrich, MO, USA) overexposure (0.1 g·L^-1^). Gill arches were dissected and live adult parasites were retrieved under the stereomicroscope. Adult specimens are easily distinguishable by having more than six pairs of clamps and vitellaria, and their location at the distal end of gill filaments. Worms (*n* = 5) with internalized fresh bloodmeal (figure 1) were chosen to quickly apply the *intra vitam* haemochromogen crystal assay [26], for haem group confirmation. A few drops of the haemochromogen crystal assay reagent (5 ml saturated glucose solution, 5 ml 10% sodium hydroxide solution, 5 ml pyridine (Sigma-Aldrich, MO, USA) and 10 ml distilled water) were applied onto worms on a slide, which were observed under light microscopy. Additionally, two positive controls were performed with fresh host blood drawn from caudal vessels using heparinized syringes. In the first positive control, a blood smear was prepared and directly treated with the haemochromogen crystal reagent. In the second, a smear of lysed peripheral blood cells (PBCs) was treated with the reagent. For this, washed PBCs were mechanically lysed in a Bullet Blender ® (Next Advance, NY, USA) with a combination of 0.5 and 0.15 mm zirconium oxide beads, centrifuged 30 s at 3,500 × *g*, and the supernatant used for the smear.

### (c) Blood intake

A modified method of Ogawa *et al*. [14] was applied to demonstrate and quantify blood intake by *S. chrysophrii*. Briefly, 10 ml of Fluoro-Max™ green fluorescent polymer microspheres (FPMs; Thermo Scientific™, Fremont, CA, USA) (1.81×10^10^ FPMs·ml^–1^) were washed in Hank’s Balanced Salt Solution (HBSS), centrifuged 5 min at 7000 × *g* and resuspended to the same volume. Infected fish (*n* = 7) were sedated in eugenol (30 ppm; Guinalima, Valencia, Spain). First, blood (100 µl) was withdrawn from the caudal vessels to determine haemoglobin, haematocrit and circulating erythrocyte (RBC) count. Then, FPMs (2 µl per body weight g) were injected into the fish bloodstream. Fish were individually allocated in 100 L sea-water tanks. At 3 h post-injection (hPI), fish were sedated and blood (100 µl) was withdrawn for RBC and FPM counts. At 18 hPI, fish were euthanized as before, blood was withdrawn to perform RBC and FPM counts and all gill arches were excised. RBC and FPM counts were performed using a haemocytometer at 312.5 X, and the FPMs·RBCs^–1^ coefficient was calculated to assess the efficiency of the FPMs' perfusion in the fish vascular system. Gill arches were inspected under the stereomicroscope and worms (*n* = 98) manually detached. Fish biometry, blood values before FPM injection and parasite numbers are detailed in table 1. Worms were treated in ethanol 70% (*v/v*) at 38°C for 1 min, preventing body contraction, and rinsed in HBSS to remove external FPMs.

**Table 1.** Biometrical and haematological (Haemoglobin = Hb; Haematocrit = Hct) values of sampled *Sparus aurata* before fluorescent polymer microsphere injection and number of retrieved adult *S. chrysophrii*.

fish	weight (g)	length (cm)	Hb (g·dL^–1^)	Hct (%)	retrieved adult parasites
**1**	147.0	18.0	3.5	31	8
**2**	134.5	17.6	3.5	22	3
**3**	557.0	28.0	3.1	19	10
**4**	125.0	17.5	5.7	35	15
**5**	103.0	16.0	3.1	21	4
**6**	216.0	20.7	9.1	51	9
**7**	162.5	18.4	2.9	19	49

Since few FPMs were detected inside the worms under a BX51 fluorescence microscope (Olympus Corporation, Tokyo, Japan), parasites were individually placed in Eppendorf tubes® (Eppendorf, Hamburg, Germany) containing 10 µl of lysis buffer (5M NaCl, 1M Tris, 10% SDS, 0.5M EDTA, pH8) and 10 µl proteinase K, and digested overnight at 38°C in agitation. Ingested FPMs were counted from the digestion product of each lysed parasite under fluorescence microscopy.

The blood volume ingested by each parasite per day was calculated from the internal FPMs and the mean circulating FMP concentration at 3 and 18 h in their fish host.

### (d) Parasite histology and histochemistry

Eighty adult worms were harvested from infected fish, fixed in Bouin’s solution and processed for routine paraffin histology. Sections (4 µm) were mounted on Superfrost™ Plus slides (Menzel-Gläser, Braunschweig, Germany) and stained with haematoxylin-eosin for anatomical observations on parasites. The LeVine *et al*. [27] method was modified for *in situ* detection of free ionic iron (Fe^3+^). Briefly, sections were deparaffinized, hydrated and endogenous peroxidase activity quenched with 0.3% H_2_O_2_ for 30 min. Slides were incubated 20 min in potassium hexacyanoferrate (II) solution with hydrochloric acid (HEMATOGNOST Fe™ staining kit, Sigma-Aldrich, MO, USA) and incubated 30 min with 3,3′-diaminobenzidine tetrahydrochloride chromogen (DAB; Sigma-Aldrich, MO, USA), then washed in deionzed water. Sections were counterstained with nuclear fast red solution, dehydrated and mounted with di-N-butyl phthalate in xylene. Negative controls were solely quenched and incubated in DAB, without HEMATOGNOST Fe™ procedure. Also, iron-depleted sections (incubated overnight in 250 µl 0.1M sodium-citrate/hydrochloric acid buffer, pH 1) were processed as described above to verify absence of Fe^3+^ staining. To identify vitellogenin and its potential associations with Fe^3+^, further sections were stained using Cleveland-Wolfe [28,29].

### (e) Transmission electron microscopy (TEM) and energy dispersive X-ray spectroscopy (EDX) TEM of the parasite digestive tract

In order to locate iron in the parasite at ultrastructural level, ten specimens were retrieved, immediately fixed in 2.5% glutaraldehyde in 0.1M sodium cacodylate buffer at pH 7.2 for 24 h at 4°C and washed in the same buffer. Sample post-fixation was performed 1 h in 1% osmium tetroxide in the same buffer, before dehydration and embedding in Spurr´s resin (Sigma-Aldrich, MO, USA). Ultrathin sections were contrasted in uranyl acetate and viewed using a Hitachi HT7800 TEM at an accelerating voltage of 120 kV (Hitachi Ltd., Tokyo, Japan).

TEM coordinates of grid sections were registered for further analysis of their elemental composition. The sites of interest were analysed by EDX TEM, using a high-resolution FEI Tecnai G2 F20 S-Twin microscope at an accelerating voltage of 200 kV (FEI, OR, USA).

### (f) Plasma Fe^2+^/Fe^3+^ levels in parasite-infected fish

To determine the effects of *S. chrysophrii* on fish-free plasmatic iron concentration, blood was withdrawn from infected (R; *n* = 28; 81 days post parasite exposure) and control (C; *n* = 23) fish with heparinized syringes, centrifuged at 2000 × *g* 10 min, and plasma was retrieved and frozen at −20°C.

Plasma Fe^2+^/Fe^3+^ levels were determined with an Iron Cromazurol kit (Linear Chemicals, Barcelona, Spain). Briefly, cromazurol reagent (100 µl) and each plasma sample (5 µl) or standard (5 µl) were placed in 96-well plates and incubated for 3 min at 37°C. Absorbances were measured at 635 nm (Infinite® M Plex; Tecan Group Ltd., Männedorf, Switzerland) and optical density (OD) was interpolated into a standard curve calculated according to manufacturer’s instructions. Two plasma sample replicates were run.

### (g) Statistical analyses

Blood intake data in the seven hosts was checked for normal distribution and equal variance and compared using the Kruskal–Wallis H-test (one-way ANOVA on ranks) with SigmaPlot v.14.5 software (Systat Software Inc., San Jose, CA, USA). The median and interquartile range were employed to calculate volume estimates of blood ingested by *S. chrysophrii*.

Generalised linear models (GLMs) with a gamma distribution were used and compared to study the relation between infection intensity (continuous predictor) and free iron concentration in plasma (response variable). Outliers were removed based on Cook’s distance (>0.5) (electronic supplementary material, figure S2). The model quality was visually assessed by analysing predictive power, variance homogeneity, influential observations, residual uniformity and its performance (electronic supplementary material, figure S3; figure S4; table S1).

Normality was assessed using Shapiro–Wilk, Anderson–Darling and Kolomogorov–Smirnov tests for both C and R groups separately and combined. Additionally, diagnostic plots, including density and quantile-quantile plots, were analysed together with the skewness, kurtosis and the equality of variances between C and R groups (Brown–Forsythe test).

Mann–Whitney *U*-test (Wilcoxon rank sum test) and Welch’s *t*‐test were used to determine differences in plasma Fe^2+^/Fe ^3+^ between R and C fish. Statistically significant differences were considered at *p*<0.05. Statistical analyses were conducted with R v.4.4.0 [30] using R packages: MASS [31], ggplot2 [32], Performance [33], Patchwork [34], DHARMa [35], car [36], Nortest [37], Moments [38], Onewaytests [39] and Dplyr [40].

### (h) Ethics statement

Experiments were carried out according to current Spanish (Royal Decree 53/2013) and EU (Directive 2010/63/EU) legislation on experimental fish handling. Procedures were approved by the Ethics and Animal Welfare Committee of the Institute of Aquaculture Torre de la Sal (IATS-CSIC, Castellón, Spain), CSIC and ‘Generalitat Valenciana’ (permit number 2021/VSC/PEA/0194).

## 3. Results

### (a) Haemochromogen crystal assay—parasite *intra vitam* observations

**Figure 2.**
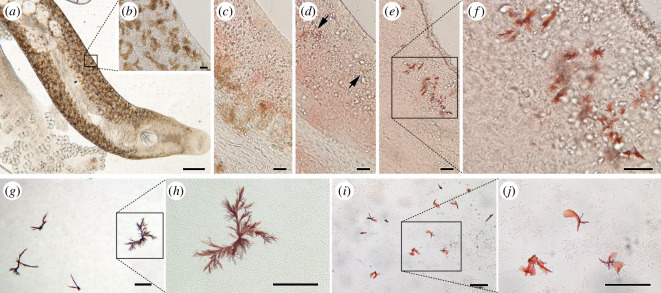
Haemochromogen crystal assay. (*a*)-(*f*) Haem group detection in *S. chrysophrii.* (*g*)–(*j*) Haem group detection in blood smears. (*a*) Wet mount of an adult specimen before reagent application. (*b*) Magnified section of the square in (*a*).(*c*) After adding the haemochromogen crystal assay reagent, it diffused along the parasite’s body, acquiring a reddish colour. (*d*) After 1 min, orange–red precipitates (black arrows) started to develop. (*e*) Red flame-like crystals developed from the precipitates in (*d*). (*f*) Magnified section of the framed box in (*e*). (*g*) Red precipitates in *S. aurata* whole blood smears. (*h*) Magnified section of the square in (*g*). (*i*) Red precipitates in mechanically lysed *S. aurata* peripheral blood cells. (*j*) Magnified section of the framed box in (*i*). Scale bars: *a* = 200 µm, *b*–*f* = 20 µm, *g*–*j* = 100 µm.

Parasites with internalized fresh blood showing the bloodmeal along their digestive system (figure 1) were used for the haemochromogen crystal assay. A few seconds after adding the reagent, pyridine haemochromogen orange/red precipitate started to develop, and in approximately 1 min, the characteristic typical flame-like pyridine ferroprotoporphyrin crystals precipitated and polymerized. The crystals were located along the parasite’s body, adjacent to the digestive caeca and the pigmented haematin cells, indicating the presence of residual iron–porphyrin complexes, also known as haem-groups (figure 2). In the first positive control using *S. aurata* blood smears, it took about 2 h for the flame-like pyridine ferroprotoporphyrin crystals to precipitate, whereas it took about 30 min for the mechanically lysed PBC smears.

### (b) Blood intake

**Figure 3.**
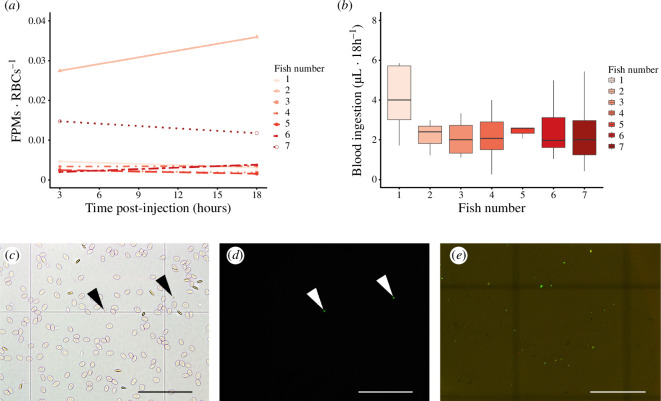
Fluorescent polymer microspheres (FPMs) count. (*a*) FPMs–erythrocyte coefficient throughout the experiment for the seven fish used. (*b*) Boxplot representing the ingested blood volume by the adult *S. chrysophrii* population from each fish. (*c*) Bright-field microphotograph of a blood sample at 18 h post FPMs injection. (*d*) Fluorescence microphotograph of the same field as in (*c*). Note the presence of two FPMs (arrow heads). (*e*) FPMs present in digested parasite (bright-field + fluorescence overlap). Scale bars = 100 µm.

The FPMs·RBCs^–1^ coefficient remained stable throughout the experiment, indicating success in the FPMs' perfusion in the fish vascular system (figure 3*a*). The parasites’ bloodmeal ingestion was calculated in regard to the FPMs·RBCs^–1^ coefficient of each fish (*n* = 7; figure 3*b*). For this purpose, individual digestion of specimens (*n* = 98) enabled ingested FPM counts (figure 3*e*). Since no statistically significant differences were observed in the blood ingested by parasites in the different fish according to the Kruskal–Wallis U test (*p* < 0.05; *p* value = 0.054) (figure 3*b*), the average blood ingestion of adult worms was calculated, resulting in 2.84 ± 2.12 µl·24 h^–1^ (median ± IQR).

### (c) Detection of free ionic iron (Fe^3+^), vitellogenin and parasite histology

**Figure 4.**
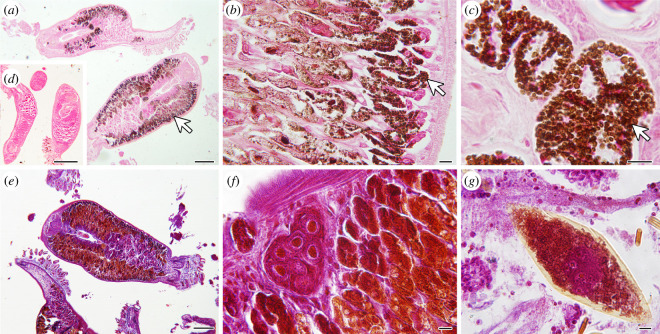
*Sparicotyle chrysophrii* microphotographs. (*a–c*) Free ionic iron (Fe^3+^) staining (brown colour; arrows) in adult specimens counterstained with nuclear fast red. (*d*) Negative control shows the absence of free ionic iron staining after iron depletion. (*e,f *) Adult specimens with vitellogenin-positive staining (orange-brown) with Cleveland–Wolfe. (*g*) Eggs with vitellogenin-positive staining with Cleveland–Wolfe. Scale bars in *a*,*d*,*e* = 200 µm; *b*,*c*,*f*,*g* = 10 µm.

Bilateral iron (Fe^3+^) deposits in *S. chrysophrii* were visible as an intense brown DAB precipitate longitudinally distributed from the rostral to caudal regions along the parasite’s digestive caeca (figure 4*a–c*). The specificity of the Fe^3+^ labelling was confirmed by the absence of staining after iron depletion (figure 4*d*). Moreover, the Cleveland–Wolfe stain revealed that the main Fe^3+^ deposits overlapped with the location of vitellogenin (figure 4*e-g*), indicating a potential association between them.

### (d) Anatomy of the parasite buccal complex

**Figure 5.**
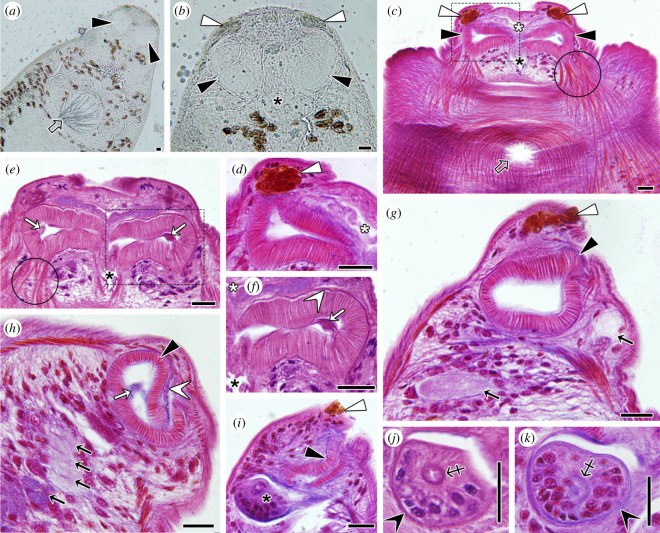
Light microscopy of the buccal complex of *Sparicotyle chrysophrii*. (*a,b*) Live specimen with two oval subterminal buccal suckers (black arrowheads), taste organs (white arrowheads) and central pharynx (black asterisk). Note the male genital atrium in ventral central position (hollow arrow) and the complex network of nerves and ducts occupying the cephalic region. (*c*) Ventral surface section at longitudinal transversal orientation showing both buccal suckers (black arrowheads), taste organs (white arrowheads), mouth cavity (white asterisk) and pharynx lumen (black asterisk). Note the prominent muscle fibres, especially the fan-shaped fibres binding to the radial muscles of the suckers (circle). The gonopore appears in a central position (hollow arrow). (*d*) Magnified section of the dashed square in (*c*). (*e*) Longitudinal transversal section across the buccal suckers showing the pharynx lumen (black asterisk) and fan-shaped muscle fibres (circle). Note the septum across each sucker lumen (white arrows). (*f*) Magnified section of the dashed square in (*e*), showing the mouth cavity (white asterisk) and the fibrous capsule around the buccal sucker (white arrowhead). (*g*) Longitudinal sagittal section across the taste organ (white arrowhead) and buccal sucker (black arrowhead). Note the presence of putative giant glandular cells with light or coarse cytoplasm (black arrows). (*h*) Buccal sucker (black arrowhead) surrounded by a fibrous capsule (white arrowhead) and with a septum across the lumen (white arrow). Note the presence of numerous glandular cells with different cytoplasm contents (black arrows). (*i*) Cephalic section across taste organ (white arrowhead), buccal sucker (black arrowhead) and pharynx (black asterisk). Note the slightly off-centred pharynx lumen. (*j, k*) Detail of pharynx cross-sections surrounded by a fibrous capsule (black arrowheads) containing numerous cells and the pharynx convoluted lumen inside a fibrous wall (cross-arrow). Stainings: Cleveland-Wolfe (*c*,*d*,*g*,*h*,*i*,*k*); haematoxylin-eosin (*e*,*f*,*j* ). Scale bars = 20 µm.

The foregut of *S. chrysophrii* is located subterminally at the anterior end. It is constituted by the mouth cavity, two lateral buccal suckers and the pharynx, placed centrally behind the mouth cavity (figure 5). A complex network of nerves and ducts, the latter probably excretory, can be distinguished in the anterior cephalic region (figure 5*a,b*). Behind the pharynx, the digestive tract bifurcates around the male copulatory organ and gonopore into the two main digestive caeca, which run laterally along the parasite’s body. The paired buccal suckers are formed by radial muscle fibres (figure 5*d–h*). Depending on the orientation and depth of the section, a septum can be observed in the lumen of the suckers linking their facing muscles (figure 5*e, f, h*). Although suckers appear subcircular in fresh specimens, in histological sections the entire annulus is rarely seen, probably because of their folded shape and angled position. Anteriorly to the buccal suckers, two refringent slightly protruding papillae-like structures were observed, which were intensely orange-stained with Cleveland–Wolfe (figure 5*b–d*,*g*,*i*). Also, some cells resembling glandular cells, with an enlarged and granular or coarse cytoplasm, were observed close to the mouth cavity and the pharynx (figure 5*g,h*). The pharynx is located posteriorly and slightly dorsally of the suckers. In cross-section, a slightly off-centred pharyngeal canal was distinguished with a convoluted lumen inside a fibrous tube (figure 5*i–k*). The buccal suckers and the pharynx are each located in a fibrous capsule surrounding them (figure 5*f,h*,j,k). Fan-shaped muscle fibres inserting buccal suckers were evident (figure 5*c,e*).

### (e) Transmission electron microscopy (TEM) and energy dispersive X-ray spectroscopy (EDX) TEM of the parasite digestive tract

**Figure 6.**
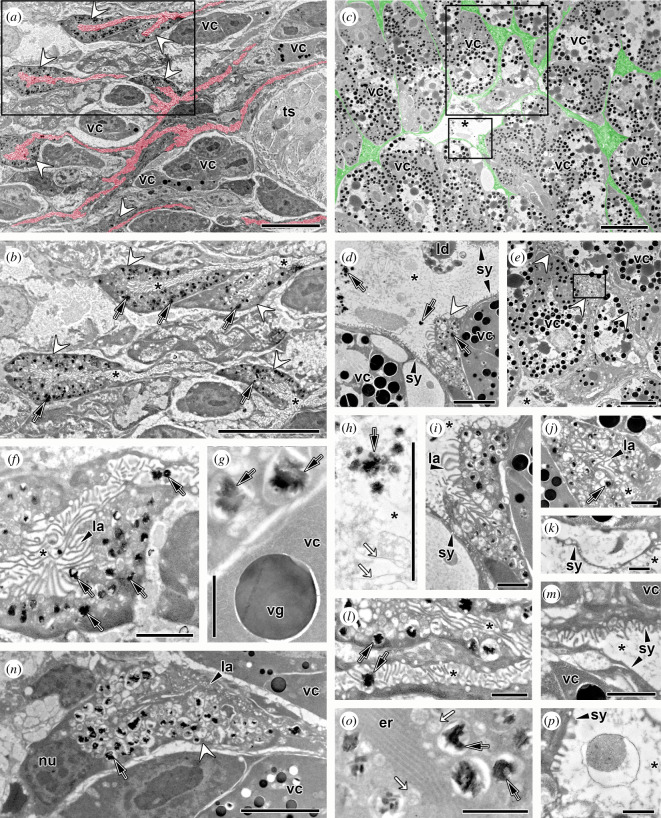
Transmission electron microscopy of *S. chrysophrii* gastrodermis. (*a*) Gut lumen (highlighted in red) lined by a syncytium interconnecting the pigmented digestive cells (haematin cells). Vitelline cells (mature and immature) and testis can be observed close to the gastrodermis. The framed box indicates the area magnified in (*b*). (*b*) Haematin cells forming gut diverticula. Note their abundant electron-dense inclusions and lamellar projections into the gut lumen. (*c*) Haematin cells and the connecting syncytium (highlighted in green) branching in between vitelline cells in vitelline follicles. Note the broad gut luminal opening in the centre. The small framed box indicates the area magnified in (*d*); the larger framed box indicates the area magnified in (*e*). (*d*) Portion of the gut lumen lined by the connecting syncytium and haematin cell. Note the presence of intracellular haematin crystals inside lysosomal vesicles and extracellular haematin crystals in the gut lumen, together with lipid-like droplets and undifferentiated debris. (*e*) Three haematin cells between vitelline cells in vitelline follicles. The framed box indicates the area magnified in (*j*). (*f*) Haematin cell with abundant lysosomal vesicles, many containing haematin inclusions. Haematin crystals are also present in the gut lumen, together with numerous branched and unbranched lamellae projecting from the haematin cell. (*g*) Haematin crystals inside lysosomal vesicles in a digestive cell close to a vitelline cell with vitelline granule. (*h*) Free haematin aggregates in the gut lumen together with some fibrous and coarse material (white arrows). (*i*) Haematin cell with abundant digestive lysosomes, haematin granules and apical lamellae. Note the continuity between the haematin cell and the connecting syncytium with shorter luminal lamellae. (*j*) Haematin cell forming a gut diverticulum full of lamellar projections. (*k*) Connecting syncytium forming balloon-like structure enclosing luminal contents. (*l*) Confluence of digestive channel with luminal haematin crystal and caecum formed by haematin cell with abundant lysosomal vacuoles, some containing haematin crystals. Note the continuity between the haematin cell and the connecting syncytium. (*m*) Connecting syncytium overlying vitelline cells. Note the upper syncytial layer with some electron-lucent vesicles and abundant short lamellae projected into the gut lumen, in contrast to the smooth syncytial layer lining on the lower vitelline cell. (*n*) Cross-section of haematin cell including the nucleus at basal position, some apical lamellae and abundant cytoplasmic vesicles, many containing haematin crystals. (*o*) Haematin cell cytoplasm with lysosomal vesicles with and without (white arrows) haematin crystals and endoplasmic reticulum. (*p*) Gut lumen with membrane-bound vacuole containing coarse undifferentiated material. White arrowheads = haematin cells; black arrows = haematin crystals; asterisk = gut lumen; er, endoplasmic reticulum; la, luminal lamellae;ld, lipid-like droplets; nu, nucleus; sy, syncytium; ts, testis; vc, vitelline cells; vg, vitelline granule. Scale bars: *a*,*b*,*e* = 10 µm; *c* = 20 µm; *d*,*h*,*n* = 5 µm; *f*,*i*,*j*,*m* = 2 µm; *g*,*k*,*l*,*o*,*p* = 1 µm.

The digestive tract of *S. chrysophrii* adult specimens presented numerous branched caeca extending between vitellaria and testis (figure 6*a–c*). The ultrastructure of the gastrodermis lining the gut lumen consisted of cup-shaped digestive cells, the haematin cells, interconnected by a digestive syncytium. Continuity between haematin cells and the digestive syncytium was noted (figure 6*i, l*). The haematin cells had a basal nucleus and an apical opening, and their cytoplasm presented extensive areas of endoplasmic reticulum and numerous lysosomal vesicles with coarse material or electron-dense crystals inside, the haematin pigment (figure 6*b,f*,*g*,*n*,*o*). Free haematin aggregates were also observed in the gut lumen, together with lipid-like droplets, undifferentiated debris and membrane-bound vacuoles, but fish erythrocytes were not identified (figure 6*d,h*,*l*,*p*). The diverticula formed by the haematin cells and the connecting syncytium were mostly covered by numerous, branched and unbranched lamellar projections on the gut luminal surface, which greatly increase the absorptive surface (figure 6*f,i*,*j,l,n,p*). Also, areas with a smooth syncytial layer lining on vitelline cells were observed and some extended lamellae formed balloon-like structures enclosing luminal contents (figure 6*k,m*). The spectral analysis of the emission of electron-dense crystals in haematin cells by EDX-TEM showed iron as the main component ( electronic supplementary material, figure S1). The spectra of the haematin cell cytoplasm, vitelline cells and worm sclerites inside the clamps were also analysed, but no iron could be detected (electronic supplementary material, figure S1).

### (f) Parasite effect on fish plasma Fe^2+^/Fe^3+^ levels

**Figure 7.**
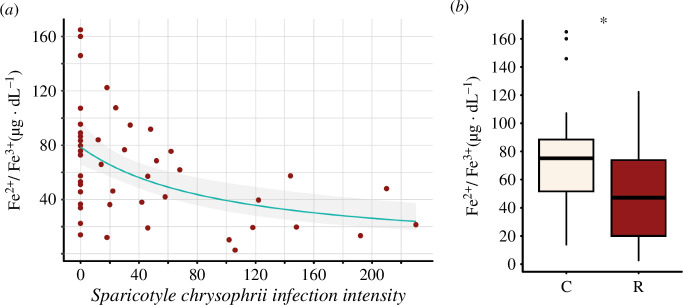
Plasmatic free ionic iron concentration in *Sparus aurata*. (*a*) Scatterplot showing the relation between the plasmatic free ionic iron concentration and *Sparicotyle chrysophrii* infection intensity. The green line represents the GLM fit on the observed data and the grey area the model’s 95% confidence intervals. (*b*) Boxplot showing plasmatic free ionic iron concentration. Asterisk indicates significant differences between C (control) and R (infected) fish (Mann–Withney U-test, Welch’s *t*‐test; *p *< 0.05).

The best gamma-distributed GLM (figure 7*a*; electronic supplementary material, table S1, figure S3) obtained after removing three outliers (one C, *n* = 22, and two R, *n* = 26) identified using Cook’s distance (electronic supplementary material, figure S2), was used to determine the effects of *S. chrysophrii* on its host’s plasmatic free iron concentration. This model met validity assumptions, including predictive power, variance homogeneity, influential observations and residual uniformity (electronic supplementary material, figure S4). Overall, the model performed well, presenting an adequate fit with akaike information criterion (AIC), AICc and bayesian information criterion (BIC) values of 472.995, 473.541, and 478.609, respectively ( electronic supplementary material, table S1), and moderate explanatory power with a Nagelkerke’s *R*^2^ ($R_{\text{N}}^{2}$) of 0.263, indicating that 26.3% of the variance in plasma free iron concentrations is explained by parasite infection intensity. The model also showed reasonable prediction accuracy, with a root mean squared error value of 33.456, and consistent reliability with a sigma value of 0.536 (electronic supplementary material, table S1 and figure S3).

The infection intensity significantly affected plasma free iron concentration, with higher parasitic burdens associated with lower iron levels, according to the Wald test (*p*<0.05; *p* value = 8.84e−04) (figure 7*a*). Normality and variance equality were checked (electronic supplementary material, figure S5, table S2). Additionally, the Mann–Whitney *U*-test (*p *< 0.05; *p* value = 0.03221) and the Welch’s *t*‐test (*p*<0.05; *p* value = 0.02197) (figure 7*b*) showed statistically significant differences between C (*n* = 22) and R (*n* = 26), providing robust evidence of the effects of these parasites on their hosts’ plasmatic free iron concentration.

## 4. Discussion

Erythrocytes are the major cellular constituent in the blood tissue, and as such, the globin moiety present in haemoglobin poses the main source of peptide and amino acid acquisition for haematophagous parasites [41,42]. Moreover, iron is indispensable for life and haem is a prosthetic group consisting of an iron atom bound to the ring of porphyrin within haemoglobin [43,44]. However, free haem is highly cytotoxic [2] and must be detoxified by haematophagous parasites [1]. In parasitic flatworms, this is accomplished by either haem breakdown by catabolic enzymes (glutathione *S*-transferase or reduced glutathione), or by the synthesis of inert haem aggregate crystals (haemozoin) [1,45–47]. An additional strategy for mitigating haem toxicity is to efficiently sequester these prosthetic groups into developing oocytes for egg formation and embryogenesis, thus regulating reproduction and ultimately increasing the iron concentration in eggs [19,20]. Haem-binding proteins involved in such process were first described in haematophagous arthropods belonging to the classes Insecta and Arachnida [40,41], but have also been identified in trematode parasites [1,23,48–51], suggesting neodermatans may also follow this strategy.

Polyopisthocotylans, including *S. chrysoprhii*, are considered haematophagous, mostly on the basis of a recognised association between high adult parasite burdens and host anaemia [52]. However, it remains uncertain to what extent the anaemia can be attributed to a direct haematophagy or to traumatic haemorrhagic lesions inflicted by the parasite’s haptor in the gill tissue.

In this study, we describe for the first time the presence of bloodmeal content in the gut of adult *S. chrysophrii* specimens (figure 1) and the *in vivo* location of haem by the haemochromogen crystal assay (figure 2). Similar results were reported in other polyospisthocotylans [26], where flame-like crystals were observed in the same location as in *S. chrysophrii*. Interestingly, the observed time differences for ferroprotoporphyrin crystal precipitation in live worms (1 min) compared to whole blood (2 h) and lysed fish blood (30 min) (figure 2) suggest the existence of a highly active haemolytic machinery in this parasite. Similar results have been reported in the polyopisthocotylan *Eudiplozoon nipponicum* Goto, 1891, the blood fluke *S. mansoni* and the hard tick *Ixodes ricinus* Linnaeus, 1758 [10,42,53]. Accordingly, large bilateral iron deposits were identified by histology along the parasite’s digestive caeca (figure 4).

TEM evidenced the structural distribution of the digestive caeca and their associated diverticula in a complex interweaving network between gonadal tissues, the male follicular testes and female vitelline follicles (figure 6). The syncytial lamellar grid lining the gastrodermis was termed the apical pinocytotic fibrous surface complex and documented in other polyopisthocotylans [17,54]. It increases the digestive surface and engulfs food material from the gut lumen into the haematin cells through membrane-bound vesicles by endocytosis. The connecting syncytium, with internal vesicles and with luminal lamellae and balloon-like structures entrapping digestive material, apparently plays an active role in nutrient absorption and distribution beyond a mere structural supporting role. The cytoplasm of adult *S. chrysophrii* haematin cells bears abundant lysosomal vesicles mostly containing electron-dense haematin crystals, and TEM-EDX revealed its main elemental component was iron (electronic supplementary material, figure S1). The lysosomal vesicles with coarse material probably contain endocytosed haemoglobin prior to digestion. Overall, this parasite’s gastrodermis morphologically resembles that of other studied polyopisthocotylans [55–59]. The luminal surface of haematin cells, with numerous vesicles and pits, suggests macromolecule uptake through endocytosis and subsequent intracellular digestion within a lysosomal system. Dalton *et al*. [41] described this so-called intracellular digestive tract, which first endocytoses haemoglobin upon a receptor-mediated selective ingestion, for monogenean blood-feeders. After the intracellular digestion of the globin moiety, the residual oxidized haem accumulates as the insoluble ferriporphyrin haematin, which is eventually extruded into the gut lumen and regurgitated. Accordingly, extracellular haematin crystals were observed in the caecal lumen of parasites, together with other undifferentiated debris.

No erythrocytes were observed in the caecal lumen of *S. chrysophrii,* suggesting that bloodmeal is haemolysed before it reaches the gastrodermis in the gut caeca for effective absorption and intracellular digestion. The absence of blood cells in the gut lumen of other polyopisthocotylans has been reported [26,41,54,56,60] and haemolysis by gland secretions in the parasite’s pharynx/foregut was suggested [17]. Giant cells, considered unicellular glands located below buccal suckers and around the pharynx, were described in other polyopisthocotylans, namely, *E. nipponicum* and *Paradiplozoon homoion* Bychowsky and Nagibina, 1959 [18,61,62], similar to our observations in the buccal complex of *S. chrysophrii* (figure 5). The released proteolytic secretions would be responsible for the extraintestinal digestion. Furthermore, the taste organ, a tegument invagination with numerous sensory receptors and glandular ducts at the anterior end of the polyopisthocotylidan *Pricea multae* Chauhan, 1945 [16], resembles the stained protrusions observed in *S. chrysophrii.* Those authors suggested that the taste organ might be protruded and directly contact the gill tissue so that its histolytic secretions would help to access surface blood capillaries. These observations are further supported by the proteomic work of Riera-Ferrer *et al*., which suggests that this parasite causes haemolytic anaemia in *S. aurata* based on a positive correlation of plasmatic alpha−1-microglobulin with infection intensity [63]. In addition, about 14% of the proteins identified in *S. chrysophrii* purified extracellular vesicles correspond to hydrolytic enzymes, including leucyl and alanyl aminopeptidases, that are thought to be involved in the final steps of haemoglobin lysis. Glutathione *S*-transferase was identified in these extracellular vesicles as well, evidencing the participation of extracellular vesicles in biological processes such as haem detoxification and Fe transport [64].

Overall, the buccal complex of *S. chrysophrii* apparently fulfils a triple function during blood feeding: sensory, attachment and haemolysis. The muscle action of buccal suckers assists in attachment of the anterior end to the feeding area. The prominent body musculature bound to the capsules holding buccal suckers and the pharynx allows them to be everted during sucking and the fan-shaped muscle fibres have an opener and stretcher function for the buccal suckers, as previously described for *E. nipponicum* and *P. homoion* [61,62]. The septum—a transverse band of musculature in the lumen of buccal suckers [62]—has been suggested to generate negative pressure contributing to attachment and sucking in other polyopisthocotylans [17].

Iron is an essential micronutrient for sustaining life. It is generally thought that haem-auxotrophic blood-feeding parasites have developed haematophagy as an alternative strategy for iron acquisition; however, not all acquired bioavailable iron originates from haemoglobin-derived haem. Previous studies in the tick *I. ricinus* have demonstrated that (i) feeding and oviposition do not rely on the intake of host blood haemoglobin, yet it is indispensable for embryogenesis; (ii) haemoglobin is an indispensable source of haem, but not a source of iron; (iii) haemoglobin-derived haem is transported from the guts to the ovaries; and (iv) vitellins are the major haem-binding proteins in the ovaries [65]. Although these aspects are largely unexplored for most haematophagous parasitic helminths, recent work on the transcriptome and proteome of *E. nipponicum* has demonstrated an abundance of ferritin transcripts, which suggests an interesting parallelism with ticks, possibly associated with blood-feeding strategy [10].

Functional studies in *S. mansoni* have shown that haem and iron are key for growth, sexual maturity, fecundity, oogenesis, as well as egg deposition and viability [23,48]. Recently, Toh *et al*. [23] described that haem could be taken into the ovary and vitellaria by transmembrane haem transporters and in the trematode *Fasciola hepatica* Linnaeus, 1758 it was reported that haem-sequestering proteins could bind haem in maturing vitelline cells within vitellaria [49]. Moreover, exogenous haem trafficking and homeostasis pathways were identified using the free-living haem-auxotroph nematode *Caenorhabditis elegans* Maupas, 1900 as a model, where proteins related to haem uptake in the nematode’s intestine and haem transporter proteins trafficking haem from the intestine to developing oocytes were identified [50]. Our histological observations suggest a close association between iron deposits and vitellogenin (figure 4), indicating a critical requirement for this trace element for reproductive purposes, which could simultaneously act as a haem detoxification pathway. However, iron was not detected in vitelline cells (cytoplasm and vitelline granule) of *S. chrysophrii* by EDX-TEM ( electronic supplementary material, figure S1), possibly owing to the maturation stage of the sampled cells [49].

Our current study demonstrates a statistically significant iron impairment in hosts that is directly related to *S. chrysophrii* infection intensity (figure 7), building onto the previously reported decrease in haemoglobin levels in infected fish [25,63]. The current findings correlate with the requirements of non-haem-derived iron for metabolic processes and with the necessity of haem for reproductive purposes as described above. These lower iron levels in hosts might hint at a specific specialized ferritin machinery in this parasite working in deep iron exploitation from host plasma.

In this study, we provide further direct evidence of *S. chrysophrii* haematophagy through intravascular injection of FPMs into the fish host and their recovery from adult parasite specimens feeding on the fish for 18 h. Our results estimate an average blood intake rate by an adult worm of 2.84 ± 2.12 µl·24 h^-1^, up to 1.5 times as much as reported for *Heterobothrium okamotoi* Ogawa, 1991 [14]. Considering that blood comprises about 5% of the fish mass [66], it can be estimated that a 30 g *S. aurata* parasitized by 100 *S. chrysophrii* adults would lose 4.80–33.07% of its blood volume daily. In market-size fish (e.g. 350 g), the same infection intensity would result in a daily 0.41–2.83% blood loss. These levels of blood loss pose a severe threat to newly introduced juvenile fish in sea cages. Monthly blood extractions should not exceed 10% of the body weight [66], and become life-threatening if blood loss surpasses 30–50% of the body weight [67]. The continuous daily blood loss caused by the parasites can quickly be fatal, given that the daily blood depletion rates far exceed the safe thresholds over a shorter time span, indicating the potentially deadly impact of the parasite on its host.

In the case of *S. aurata* reaching market size, the parasite's impact would be relatively lower because fish would be able to cope with the blood loss produced by relatively high parasitic burdens, thus becoming reservoirs. In Mediterranean sea cages, the common fish farming practice overlaps different year-class groups in the same sites to cater for a wide range of harvest sizes and dates. From a health surveillance perspective, adopting an *all-in*, *all-out* system is crucial. This approach ensures that different year-class fish stocks do not cohabit in the same farming site, thereby preventing older animals from infecting newly introduced ones in the cages. Furthermore, integrating current *S. chrysophrii* control measures [13] with additional strategies to mitigate the anaemia and plasmatic iron decrease in parasitized *S. aurata* could significantly improve their health.

Iron homeostasis in fish is crucial for various biological functions, including respiration, energy metabolism, immune function, DNA synthesis and repair, detoxification and overall optimal cellular function maintenance [68]. Dietary iron requirements have been studied for different cultured species, including *S. aurata*. These studies indicate that low iron levels reduce growth performance, depress erythropoiesis and worsen heat-induced hypoxia [69–71]. However, these iron concentration values have been determined in very few cases based on serum or plasmatic iron levels. In *S. aurata*, iron supplementation enhanced the performance mainly from a haematological and immunological point of view [72]. Trials involving iron overload suggest that the iron homeostasis seems to be much more complex than in other vertebrates, probably owing to the involvement of the different mediators between iron metabolism and host immune response [73]. Therefore, future research should prioritize establishing the correlation between plasma iron levels and the health status of fish.

## 5. Conclusions

In this study, we demonstrate the presence of blood and exogenous haem groups, estimate the average volume of blood intake in *S. chrysophrii* and describe the functional anatomy of its buccal and gastrodermal apparatus. Moreover, our results suggest the necessity of an iron source other than haem for the flatworm’s survival, and an intimate relationship between blood intake, haem detoxification and parasite reproduction, compatible with strategies already described in other haematophagous parasites.

## Data Availability

Data, code and materials supporting this paper are publicly available at Zenodo repository [74]. Supplementary material is available online [75].
